# Oncogenic role of HMGA2 in fusion-negative rhabdomyosarcoma cells

**DOI:** 10.1186/s12935-020-01282-z

**Published:** 2020-05-24

**Authors:** Kazutaka Ouchi, Mitsuru Miyachi, Shigeki Yagyu, Ken Kikuchi, Yasumichi Kuwahara, Kunihiko Tsuchiya, Tomoko Iehara, Hajime Hosoi

**Affiliations:** 1 Department of Pediatrics, Graduate School of Medical Science, Kyoto Prefectural University of Medicine, 465 Kajii-cho, Hirokoji, Kamigyo-ku, Kyoto, 602-8566 Japan; 2 Department of Molecular Biochemistry, Graduate School of Medical Science, Kyoto Prefectural University of Medicine, 465 Kajii-cho, Hirokoji, Kamigyo-ku, Kyoto, 602-8566 Japan

**Keywords:** HMGA2, Fusion-negative rhabdomyosarcoma, Netropsin

## Abstract

**Background:**

Rhabdomyosarcoma (RMS) is the most common pediatric soft tissue sarcoma. There are two subtypes, fusion gene-positive RMS (FP-RMS) and fusion gene-negative RMS (FN-RMS), depending on the presence of a fusion gene, either *PAX3*-*FOXO1* or *PAX7*-*FOXO1*. These fusion genes are thought to be oncogenic drivers of FP-RMS. By contrast, the underlying mechanism of FN-RMS has not been thoroughly investigated. It has recently been shown that HMGA2 is specifically positive in pathological tissue from FN-RMS, but the role of HMGA2 in FN-RMS remains to be clarified.

**Methods:**

In this study, we used FN-RMS cell lines to investigate the function of HMGA2. Gene expression, cell growth, cell cycle, myogenic differentiation, tumor formation in vivo, and cell viability under drug treatment were assessed.

**Results:**

We found that *HMGA2* was highly expressed in FN-RMS cells compared with FP-RMS cells and that knockdown of *HMGA2* in FN-RMS cells inhibited cell growth and induced G1 phase accumulation in the cell cycle and myogenic differentiation. Additionally, we showed using both gain-of-function and loss-of-function assays that *HMGA2* was required for tumor formation in vivo. Consistent with these findings, the HMGA2 inhibitor netropsin inhibited the cell growth of FN-RMS.

**Conclusions:**

Our results suggest that HMGA2 has important role in the oncogenicity of FP-RMS and may be a potential therapeutic target in patients with FN-RMS.

## Background

Rhabdomyosarcoma (RMS) is the most frequent soft tissue sarcoma in children [1]. Two major histological subtypes are described, conventionally named embryonal RMS (ERMS) and alveolar RMS (ARMS), reflecting their morphological similarities to fetal muscle or pulmonary alveoli, respectively [2]. In the era of molecular profiling, two fusion genes have been identified in RMS: the *PAX3*–*FOXO1* gene fusion [3] and the *PAX7*–*FOXO1* gene [4]. These gene fusions are found in about 70% to 80% of histologically defined ARMS and are not found in ERMS [5, 6]. Several studies of ARMS have shown that *PAX*–*FOXO1* fusion gene-positive status is associated with worse prognosis than fusion gene-negative status [7, 8]. Furthermore, patients with fusion gene-negative ARMS have clinical outcomes as favorable as those of ERMS patients compared with fusion gene-positive ARMS, in accordance with the similarity in the molecular features between fusion gene-negative ARMS and ERMS [8]. Hence, identification of this fusion status, regardless of histological subtype, is being incorporated into future Children’s Oncology Group (COG) Soft Tissue Sarcoma protocols [9].

Several studies recently revealed that the *HMGA2* expression level is significantly higher in fusion gene-negative RMS (FN-RMS) than in fusion gene-positive RMS (FP-RMS) and that strong immunohistochemical expression of HMGA2 protein is specific to FN-RMS, suggesting that HMGA2 is a surrogate marker of fusion status in RMS [2, 9]. HMGA2 is a member of the high mobility group A (HMGA) family [10, 11]. The HMGA family protein, which contains three short basic repeats, so-called AT-hooks, binds the minor groove of AT-rich DNA sequences via their DNA-binding domain, which is located in the amino-terminal region of the protein [11]. HMGA protein itself does not have transcriptional activity. It acts as a transcriptional modulator by changing the affinity of transcriptional factors for target DNA sequences and altering chromatin structure, thereby regulating the transcriptional activity of other genes [12, 13]. However, limited information is available regarding the function of HMGA2 in FN-RMS.

Netropsin is a small-molecule protein that binds to the minor grooves of AT-rich DNA through a sequence- and conformation-dependent mechanism. Because the binding mechanism is similar to that of HMGA family protein, netropsin has been reported to compete with the HMGA family proteins HMGA1 and HMGA2 for DNA binding [14, 15].

The aim of this study was to investigate the role of HMGA2 in FN-RMS cells and the antitumor efficacy of netropsin in FN-RMS. We examined the effect of HMGA2 suppression on FN-RMS cells. A reduction in HMGA2 expression led to cell growth inhibition, cell cycle arrest, and myogenic differentiation. Furthermore, we showed that netropsin inhibited the cell growth of FN-RMS cells. These results indicate that HMGA2 represents a new candidate for the treatment of FN-RMS.

## Materials and methods

### Cell culture

FN-RMS cell lines (RD, RMS-YM, and Rh18), FP-RMS cell lines (Rh30 and RM2), mouse myoblast C2C12 cells, and human embryonic kidney HEK293 cells were cultured in high-glucose Dulbecco’s modified Eagle’s medium (DMEM) supplemented with 10% FBS, penicillin (100 U/ml), and streptomycin (10 mg/ml) at 37 °C in a humidified atmosphere containing 5% CO_2_. RD, Rh-18, Rh30 and RM2 cell lines were kind gifts from Dr. Peter Houghton (The Research Institute at Nationwide Children’s Hospital, Columbus, OH). The RMS-YM and HEK293 cell lines were obtained from RIKEN BioResource Center (Tsukuba, Japan). Mouse myoblast C2C12 cells and human embryonic kidney HEK293 were purchased from the American Type Culture Collection (Manassas, VA).

### Quantitative reverse transcription-polymerase chain reaction

Total RNA was extracted from tumor cells using the RNeasy Mini-Kit (Qiagen, Venlo, the Netherlands). cDNA was synthesized using the SuperScript VILO cDNA Synthesis Kit (Invitrogen, Basel, Switzerland). Real-time reverse transcription-polymerase chain reaction (RT-PCR) was carried out on a 7500 Fast Real-Time PCR system (Applied Biosystems, Rotkreuz, Switzerland) with SYBR Premix Ex Taq II (Takara Bio, Shiga, Japan), and relative quantitation was performed using the 2^−ΔΔCt^ method with glyceraldehyde-3-phosphate dehydrogenase (GAPDH) as the reference gene. The following primer sequences were used: HMGA2, forward primer: 5′-CCTGCTCAGGAGGAAACTGA-3′, reverse primer: 5′-CCTCTTCGGC AGACTCTTGT-3′; GAPDH, forward primer: 5′-GCACCGTCAA GGCTGAGAAC-3, reverse primer: 5′-ATGGTGGTGA AGACGCCAGT-3′. Each quantitative RT-PCR experiment was performed in triplicate, and the quantitative RT-PCR experiments were repeated two or three times.

### siRNA knockdown of HMGA2

Transient transfection assays were performed using commercially available siRNAs specific for inhibition of HMGA2 (s15616 and s194863; Life Technologies, Carlsbad, CA, USA) along with a negative control siRNA (4390843; Life Technologies) with Lipofectamine RNAiMAX (Life Technologies) according to the manufacturer’s instructions.

### Western blotting

Cells were lysed with Laemmli sample buffer. Protein concentrations in the cell lysates were measured with the Bio-Rad DC Protein Assay (Bio-Rad Laboratories, Hercules, CA, USA). Samples were boiled for 5 min in sample buffer containing bromophenol blue and 1 × β-ME, and equal amounts of protein were separated by sodium dodecyl sulfate–polyacrylamide gel electrophoresis (SDS-PAGE). Electrophoretic separation was carried out on 10% polyacrylamide gel (Bio-Rad Laboratories), and the proteins were subsequently transferred to Immobilon-P membrane (Millipore, Billerica, MA, USA). Membranes were blocked in phosphate-buffered saline (PBS) with Tween 20 (PBST) with 5% nonfat dry milk powder and incubated with the following primary antibodies: HMGA2 (1:250 dilution; sc-30223, Santa Cruz, Dallas, TX, USA) or FLAG (1:1000 dilution; F3040, Sigma-Aldrich, St. Louis, MO, USA). The primary and secondary antibodies for HMGA2 were diluted with Can Get Signal (Toyobo, Osaka, Japan). The membranes were then washed with PBST and incubated with horseradish peroxidase-conjugated goat anti-mouse or anti-rabbit secondary antibody (GE Healthcare, Little Chalfont, UK). Antibody binding was detected with the enhanced chemiluminescence detection system (ECL and ECL Plus; GE Healthcare).

### Cell growth analysis

Cells were plated in normal growth medium in triplicate in 24-well plates. After 24 h, cells were transfected with HMGA2 siRNA or negative control siRNA for an additional 24 h. Then, the cells were lysed under hypotonic conditions, as described previously [16], and nuclei were counted every 48 h with a Coulter counter (ERMA Inc., Jacksonville, FL, USA) until 96 h later (day 6). All experiments were conducted in triplicate for each cell line.

### Cell cycle analysis

Cells were seeded in normal growth medium in triplicate in 12-well plates. After 24 h, cells were transfected with HMGA2 siRNA or negative control siRNA for an additional 24 h. After transfection, RD, RMS-YM, and Rh18 cells were incubated in normal growth medium for 48 h, 24 h, and 24 h, respectively. Then, cells were harvested and stained with propidium iodide (PI). PI fluorescence was read on a FACSCalibur (BD Biosciences, Franklin Lakes, NJ, USA), and the data were analyzed with Cell Quest software (BD Biosciences). The cell cycle phase was determined on the basis of DNA content using ModFit LT Software (Verity Software House, Topsham, ME, USA) as described previously [17].

### Induction of myogenic differentiation

To induce myogenic differentiation, cells were rinsed thoroughly with PBS 24 h after siRNA transfection and then cultured with DMEM containing 2% horse serum (Thermo Fisher Scientific, Waltham, MA, USA), penicillin (100 U/ml), and streptomycin (10 mg/ml). Three days later, cells were observed with a BZ-8000 confocal microscope (Keyence, Osaka, Japan) to assess morphological changes. For immunofluorescence, cells on coverslips were fixed with absolute methanol, washed, and incubated with anti-myosin heavy chain (MHC) antibody (M4276, Sigma-Aldrich) for 1 h, rinsed with PBS, incubated with fluorescein isothiocyanate-conjugated anti-mouse IgG (A-11001, Invitrogen) for 1 h, and visualized using a fluorescence microscope as described previously [18].

### Lentiviral procedures and short hairpin RNA

PLKO.1 lentiviral shRNAmir constructs were obtained from Thermo Fisher Scientific (Waltham, MA, USA; HMGA2 shRNA, RHS4533; negative control shRNA, RHS 4080). The constructs were co-transfected with the packaging construct (psPAX2) and the VSV-G envelope expression plasmid (pMD2.G), both purchased from Addgene (Cambridge, MA, USA), into 293FT cells using FuGENE 6 (Promega, Madison, WI, USA). For infection, cells were incubated with lentiviral particles and 4 µg/ml polybrene (Nacalai Tesque, Kyoto, Japan), and then selected with puromycin.

### Retroviral procedures

C2C12 cell lines stably expressing Flag-tagged HMGA2 were established using a murine stem cell virus (MSCV) retrovirus expression system (Clontech Laboratories Inc., Madison, WI, USA). Platinum-E cells were transfected in 60-mm dishes at about 50% confluence with 1 mg of purified expression vector DNA, 8 µl of Enhancer, and 7.5 µl of Effectene (Qiagen, Hombrechtikon, Switzerland) in 1 ml of high-glucose DMEM. For retroviral transduction, C2C12 cells were incubated with retroviral particles and 4 µg/ml polybrene (Nacalai Tesque). Stably transfected cells were selected with 1000 mg/ml of G418 sulfate (Life Technologies).

### In vivo tumorigenesis

To assess tumorigenesis, 2 × 10^6^ of FN-RMS or C2C12 cells were subcutaneously inoculated into the back of 4-week-old athymic nude mice (BALB/c nu/nu; Shimizu Laboratory Supplies, Kyoto, Japan). Tumor diameter was monitored every 2 or 3 days after the onset of tumor formation. The mice were killed when the tumor size reached 17 mm in diameter. The mice used for this study were handled in strict adherence with local governmental and institutional animal care regulations. All studies involving mice were performed using protocols approved by the Animal Investigation Committee of Kyoto Prefectural University of Medicine.

### Cell viability assay

WST-8 colorimetric assays were carried out using Cell Count Reagent SF (Nacalai Tesque). RD, RMS-YM, and Rh18 cells were seeded in 96-well plates at 1 × 10^4^ cells/well, 10 × 10^4^ cells/well, and 8 × 10^4^ cells/well, respectively, in 100 µl culture medium per well. After 24 h, the cells were treated for an additional 96 h with netropsin (Enzo Life Sciences, Farmingdale, NY, USA) dissolved in H_2_O. Cell viability was determined colorimetrically by the optical density at a wavelength of 450 nm using a microplate reader (Multiscan JX; Dainippon Sumitomo Pharmaceutical, Osaka, Japan) as previously described [19].

### Statistical analysis

Data are shown as the mean ± SEM. Single-group data were assessed using the Student’s *t* test. The Tukey–Kramer test was performed for multiple comparisons. P-values less than 0.05 were considered to represent statistically significant differences.

## Results

### Overexpression of HMGA2 in FN-RMS cell lines

**Fig. 1:**
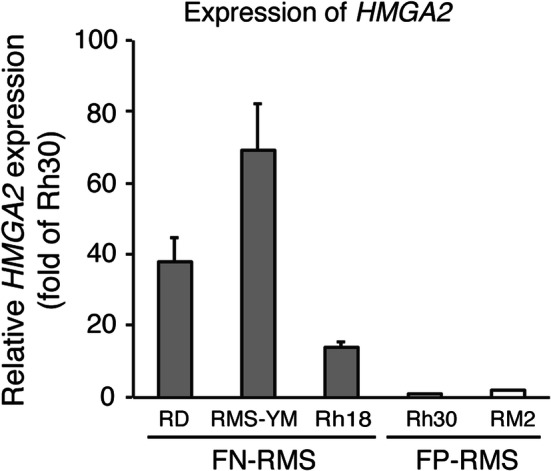
*HMGA2* expression in FN-RMS and FP-RMS cells. FN-RMS cells (RD, RMS-YM, and Rh18) and FP-RMS cells (Rh30 and RM2) were harvested and total RNA was extracted. The mRNA level for HMGA2 was measured by quantitative RT-PCR and normalized against the level of GAPDH mRNA. Columns, mean of two independent experiments; bars, SD

First, we checked the expression of *HMGA2* in the FN-RMS cell line. As expected, HMGA2 mRNA was highly expressed in FN-RMS cell lines compared with FP-RMS cell lines (Fig. 1).

### siRNA knockdown of HMGA2 inhibits FN-RMS cell growth

**Fig. 2:**
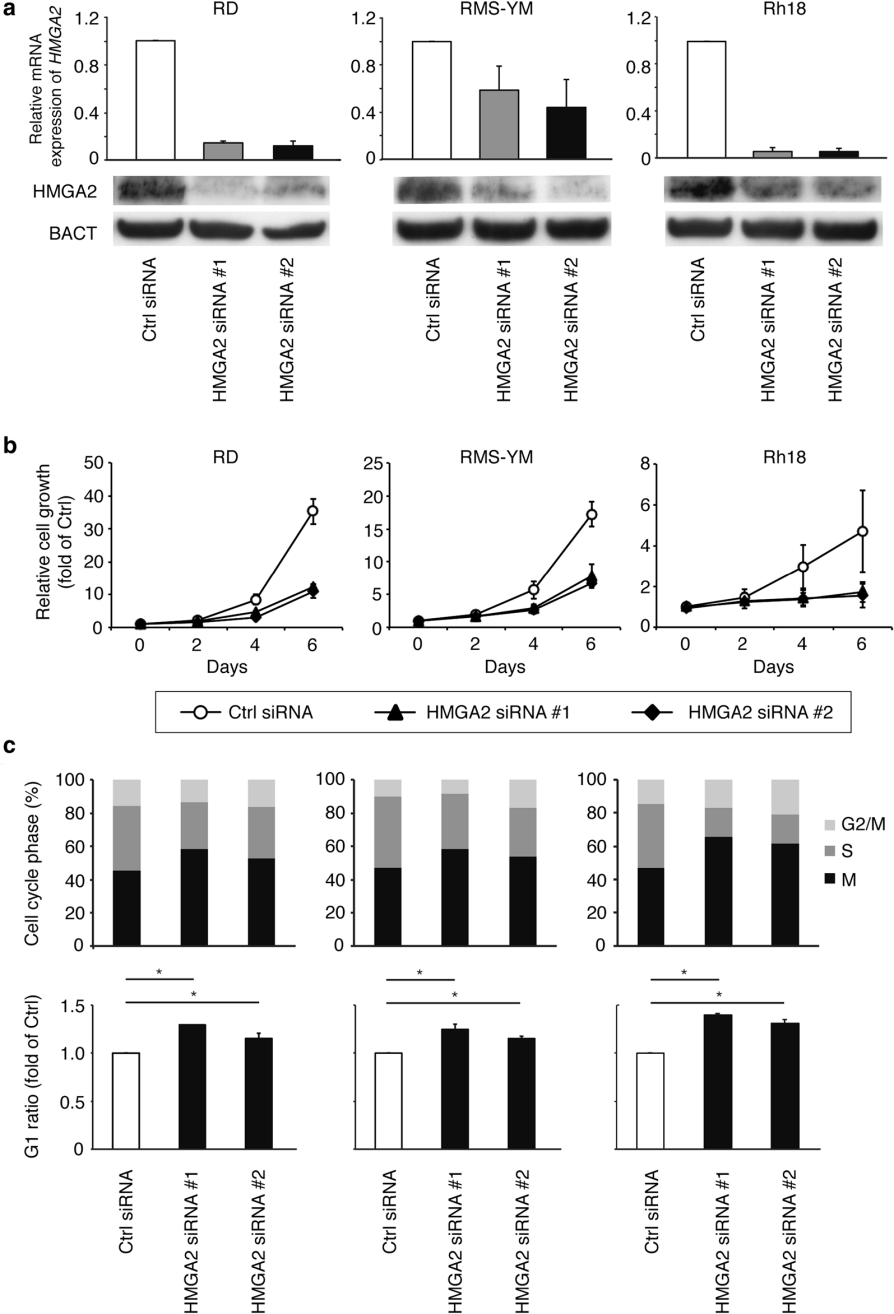
Effect of HMGA2 siRNA knockdown on FN-RMS cell growth. **a** RD, RMS-YM, and Rh18 cells were transfected with HMGA2 siRNA or negative control siRNA. Expression of HMGA2 was measured by quantitative RT-PCR and immunoblotting. Columns, mean of three independent experiments; bars, SD. * indicates statistical significance (P < 0.01; Student’s t-test). **b** Cell growth assay. RD, RMS-YM, and Rh18 cells were seeded in 24-well plates, cultured for 24 h, and then incubated with HMGA2 siRNA or negative control siRNA (day 0). Cells were harvested every 48 h, and nuclei were counted. Points, mean of three independent experiments; bars, SD. * indicates statistical significance (P < 0.01; Student’s t-test). **c** Cell cycle analysis was carried out by using siRNA-treated FN-RMS cells. RD, RMS-YM, and Rh18 cells were harvested and stained with propidium iodide and then analyzed for DNA content with FACSCalibur after siRNA transfection for 48 h, 24 h, and 24 h, respectively. Results represent the mean ± SD of three independent experiments (P < 0.01; Student’s t-test)

We next examined the biological function of HMGA2 in FN-RMS cells by using the siRNA knockdown approach. The knockdown specifically reduced HMGA2 mRNA and protein expression in RD, RMS-YM, and Rh18 RMS cells (Fig. 2a). Knockdown of HMGA2 induced cell growth inhibition in these cells, whereas control siRNA did not affect cell growth (Fig. 2b).

### siRNA-mediated HMGA2 reduction results in G1 phase accumulation

To examine the effect of the HMGA2 reduction on the cell cycle, FACS analysis was performed. As shown in Fig. 2c, HMGA2 siRNA-treated cells exhibited a significantly higher proportion of cells in G1 (siRNA #1: 58.4 ± 0.8%, 58.6 ± 4.9%, and 65.5 ± 2.8%; siRNA #2: 52.1 ± 2.6%, 54.1 ± 1.0%, and 61.3 ± 2.9%, respectively) compared with control cells (45.2 ± 0.5%, 47.0 ± 2.0%, and 46.7 ± 2.3%, respectively) in RD, RMS-YM, and Rh18 cells. The G1 ratio was statistically higher in all cell lines compared with control (P < 0.01). These data suggest that HMGA2 reduction leads to cell cycle arrest in G1.

### siRNA knockdown of HMGA2 induces myogenic differentiation of FN-RMS cells

**Fig. 3:**
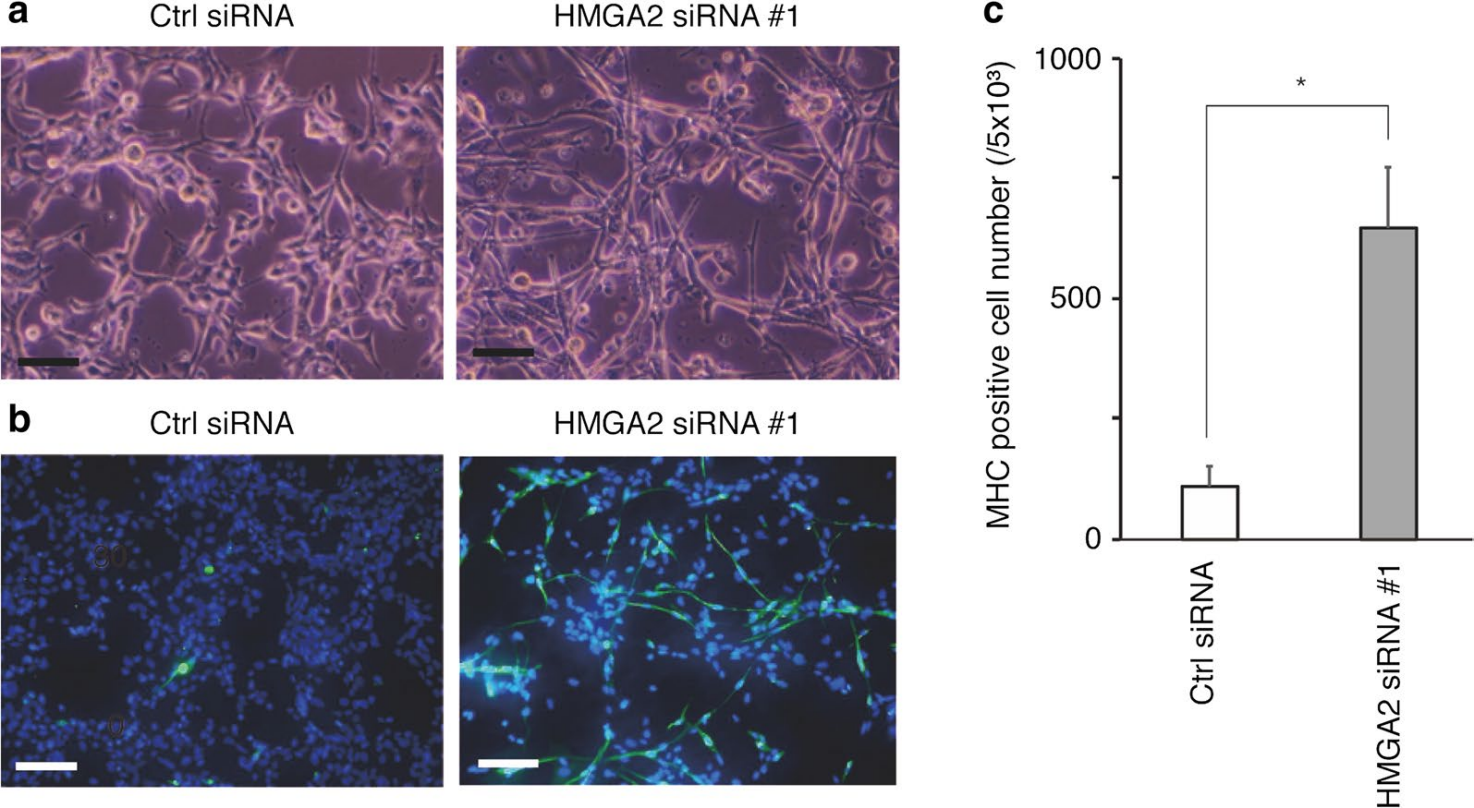
Derivation of myogenic differentiation by HMGA2 siRNA knockdown. **a** Representative light microscopy images of HMGA2-depleted RD cells and control RD cells after 72 h in differentiation medium. Scale bar, 100 mm. Some HMGA2-depleted RD cells formed myotubes. **b** Fluorescent images of MHC staining after 72 h in differentiation medium. Scale bar, 50 mm. Representative images of HMGA2-depleted RD cells showing MHC and DAPI (for nuclei), whereas control RD cells showed few MHC-positive cells. **c** Numbers of MHC-positive cells per 5.0 × 10^3^ cells. Results represent the mean ± SD of three independent experiments. *P < 0.01 compared with control RD cells

After siRNA transfection, RD cells were cultured for 72 h in differentiation medium. HMGA2 reduction morphologically promoted myotube differentiation (Fig. 3a). Cells were then stained with the antibody for the MHC, a marker of myogenic differentiation (Fig. 3b). The MHC positivity rates of HMGA2-reduced cells and control cells were 12.9 ± 2.6% and 2.2 ± 0.8%, respectively (P < 0.01; Fig. 3c). These data show that HMGA2 inhibits muscle differentiation.

### HMGA2 knockdown inhibits tumor growth of FN-RMS cells in vivo

**Fig. 4:**
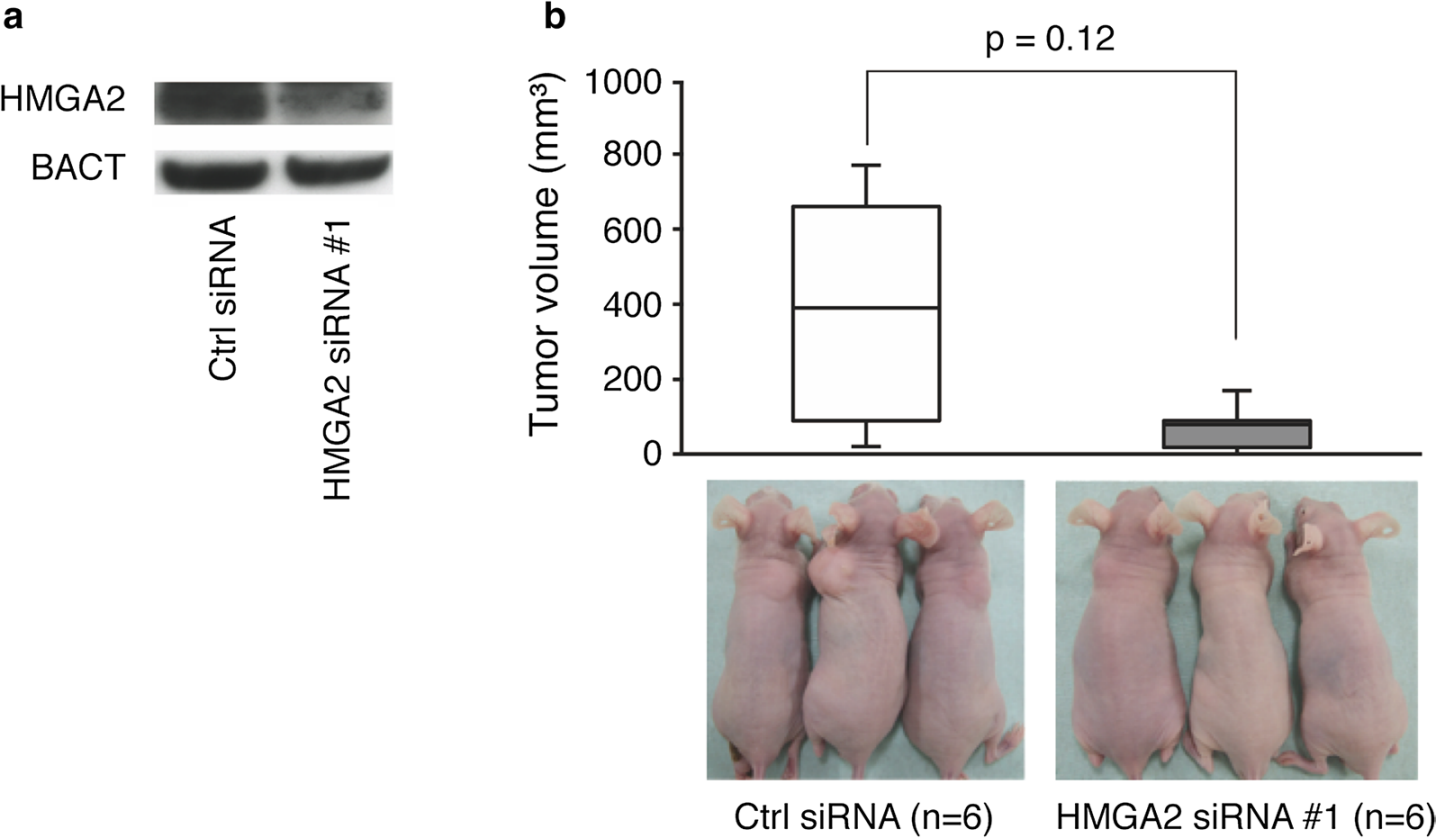
Effects of HMGA2 knockdown on tumor growth in a murine xenograft model. **a** Expression of HMGA2 as measured by immunoblotting. **b** The upper figure shows the tumor volume 9 weeks after injection of RMS-YM cells into the back of nude mice (n = 6). Data are shown by a box plot. The lower images are representative photos of mice

To assess the effect of loss of function of HMGA2, we established RMS-YM cell lines in which HMGA2 was stably knocked down using lentiviral vectors encoding HMGA2 shRNA. Western blot experiments confirmed that transduced cells expressed lower levels of HMGA2 protein than non-transduced cells (Fig. 4a). These cells were transplanted subcutaneously into nude mice. Nine weeks after injection, the tumor volume was 80.7 ± 89.9 mm^3^ for HMGA2-expressing cells but 396.3 ± 359.4 mm^3^ for control cells (P = 0.12; Fig. 4b).

### Ectopic HMGA2 expression promotes tumorigenesis of C2C12 in vivo

**Fig. 5:**
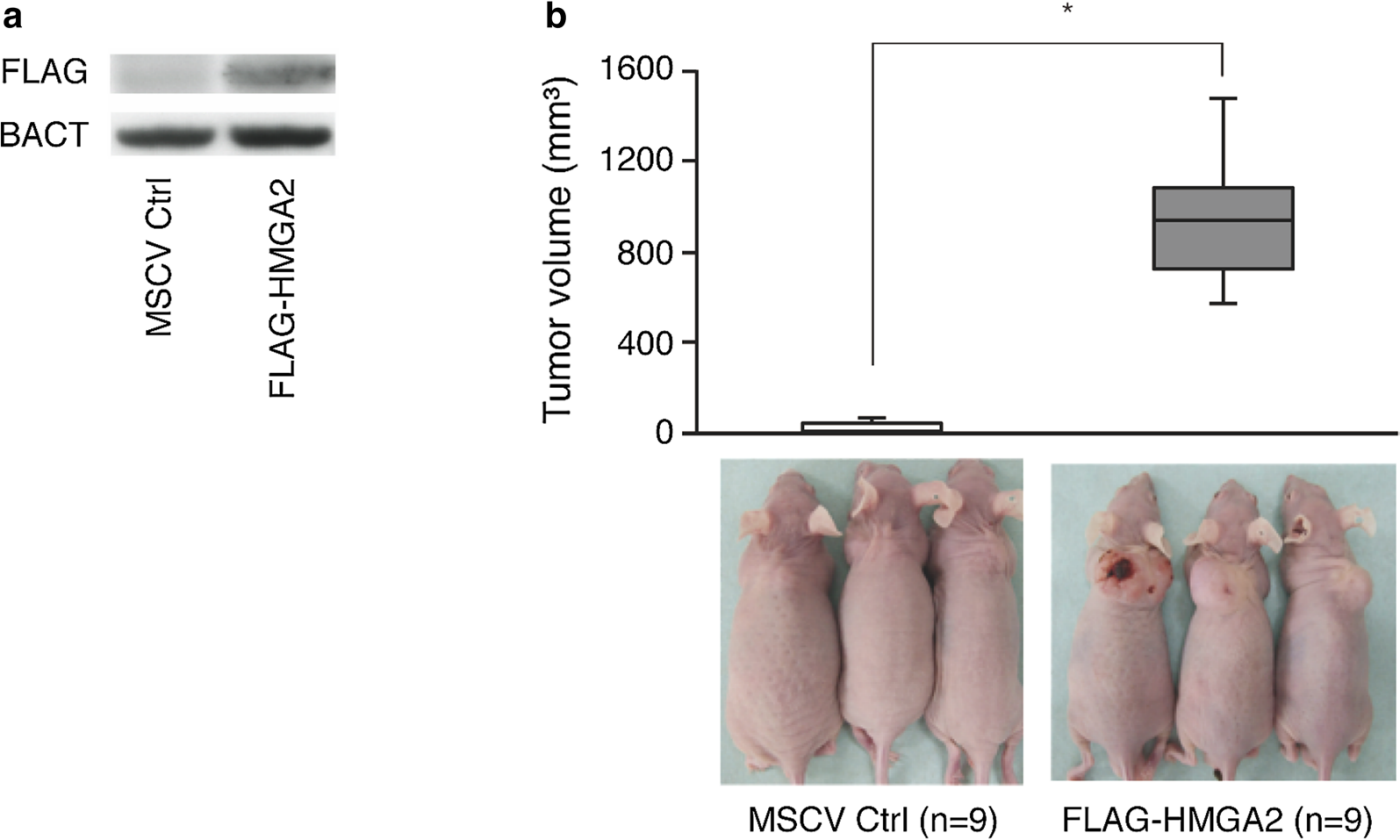
Effects of HMGA2 on the proliferation of C2C12 cells in a murine xenograft model. **a** Expression of FLAG as measured by immunoblotting. **b** The upper figure shows the tumor volume 7 weeks after injection of C2C12 into the back of nude mice (n = 9). Data are shown by a box plot. *P < 0.01 compared with control C2C12 cells. The lower images are representative photos of mice

We established Flag-tagged HMGA2-expressing C2C12 cells using MSCV retroviral systems; induction of Flag-tagged HMGA2 protein expression in the transfected C2C12 is shown in Fig. 5a. These cells were harvested and transplanted into nude mice to allow tumor formation. Seven weeks after transplantation, the average tumor volume was 970.2 ± 476.9 mm^3^ for HMGA2-overexpressing C2C12 cells but 27.2 ± 28.4 mm^3^ for control C2C12 cells (P < 0.01; Fig. 5b).

### Netropsin inhibits growth of FN-RMS cells

**Fig. 6:**
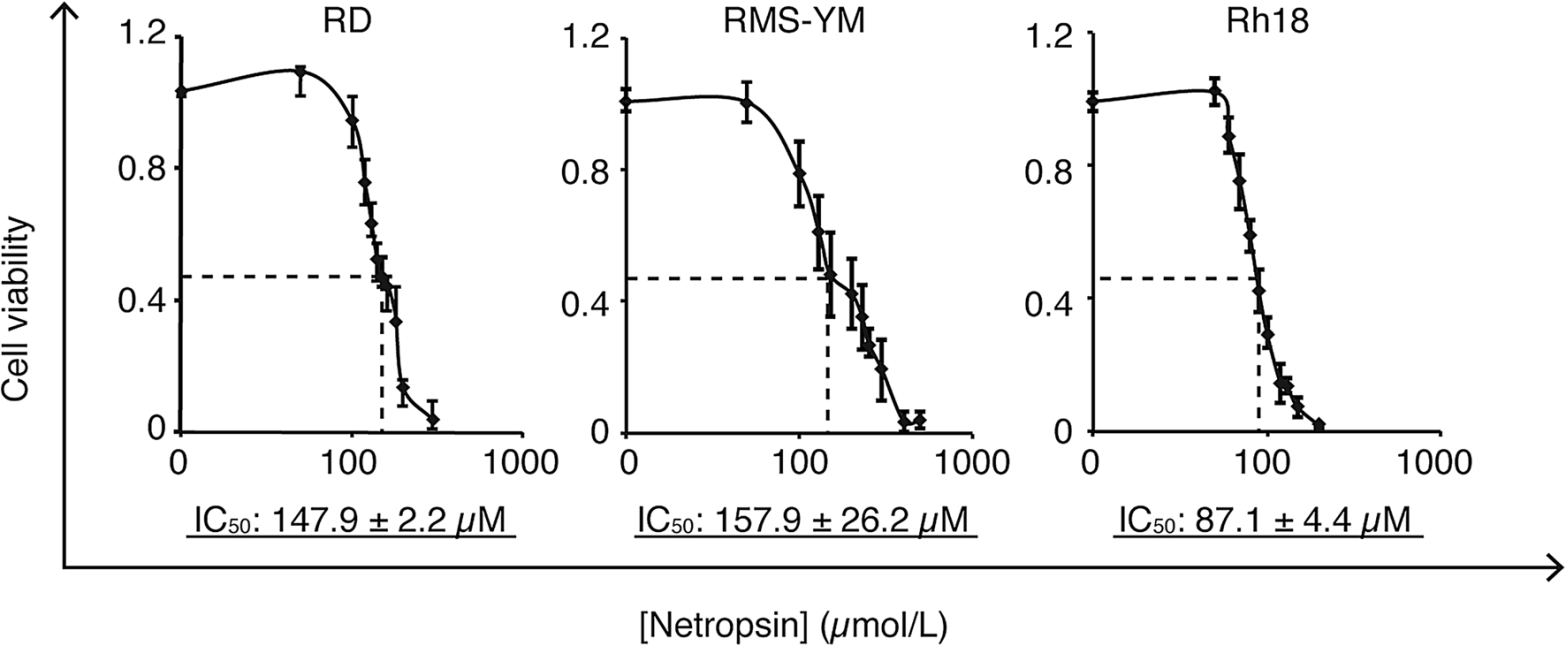
Cytotoxic effects of netropsin. RD, RMS-YM, and Rh18 cells were seeded in 96-well plates, incubated for 24 h, treated with netropsin (10–500 µM), and then analyzed. The IC_50_ values of netropsin for RD, RMS-YM, and Rh18 cells were 47.9 ± 2.2, 157.9 ± 26.2, and 87.1 ± 4.4 µM, respectively

Given that HMGA2 depletion resulted in cell growth (Fig. 2b), we hypothesized that netropsin could inhibit FN-RMS cells by competing with the HMGA2–DNA minor groove interaction [14]. When FN-RMS cells were treated with netropsin at concentrations of 10–500 µM, the proliferation was inhibited in a dose-dependent manner. In this assay system, the IC_50_ values were 147.9 ± 2.2, 157.9 ± 26.2, and 87.1 ± 4.4 µM in RD, RMS-YM, and Rh18 cells, respectively (Fig. 6).

## Discussion

The purpose of this study was to investigate the oncogenic role of HMGA2 in FN-RMS cells and the antitumor effect of netropsin on FN-RMS. Indeed, not only did HMGA2 suppression repress tumor genesis, but ectopic HMGA2 expression promoted tumorigenesis in our in vivo model. Administration of netropsin inhibited tumor cell growth.

Various insights have been provided into FP-RMS [20] and PAX3-FOXO1 is thought to be responsible for their malignant phenotypes [21]. In contrast, the underlying oncogenic factor of FN-RMS has not been fully elucidated [22]. Given that HMGA2 is highly expressed specifically in FN-RMS [2], we investigated whether HMGA2 plays an oncogenic role in FN-RMS. *HMGA2* is widely expressed during embryogenesis [23, 24] but is not observed in adult human tissues [24]. Low expression of HMGA2 has been reported in only undifferentiated cells, such as human CD34-positive hematopoietic stem cells [25], mouse preadipocyte cells [26], or meiotic and postmeiotic cells [27, 28]. A high expression of *HMGA2* has been observed in pancreatic carcinomas [29], non-small cell lung carcinomas [30], and squamous carcinomas of the oral cavity [31]. Moreover, *HMGA2* expression is associated with a more malignant phenotype and a poor prognosis in squamous carcinomas of the oral cavity [31], nasopharyngeal carcinomas [32], glioblastoma [33], esophageal squamous carcinoma [34], lung cancer [35], and atypical teratoid/rhabdoid tumor [36]. We demonstrated that *HMGA2* reduction inhibited FN-RMS tumor growth in vivo (Fig. 4) and that ectopic *HMGA2* expression resulted in tumor development in vivo (Fig. 5). These results show the oncogenic role of HMGA2 in FN-RMS. The reason for the high *HMGA2* expression in FN-RMS remains unclear. One possible mechanism is a genomic gain or amplification. Indeed, a genomic gain or amplification was observed in the region of chromosome 12q13-15, which is the locus of *HMGA2*, in FN-RMS [37, 38]. Another possible mechanism is a position effect due to a chromosomal rearrangement. Storiazzi et al. investigated a case of polycythemia vera with *HMGA2* gene rearrangement and found that the upregulation of the *HMGA2* transcript was very likely due to a position effect [39].

As the underlying mechanism of the oncogenesis, the effects of HMGA2 on cellular proliferation, invasion, the epithelial–mesenchymal transition, and apoptosis inhibition have been reported in various tumor cells [34, 40–45]. Cai et al. [42] showed that dysregulated HMGA2 contributed to cellular proliferation through cell cycle progression in prostate cancer. Our study showed that downregulation of HMGA2 led to cell cycle arrest and inhibited cell proliferation in FN-RMS (Fig. 2b, c). These results indicate that high HMGA2 expression causes cell cycle upregulation and cell growth. Moreover, in our study, the HMGA2 reduction induced myogenic differentiation of FN-RMS cells (Fig. 3). In the skeletal muscle lineage, HMGA2 expression is high in proliferating skeletal myoblasts and is significantly reduced with muscle differentiation [46]. RMS displays a myogenic phenotype with the expression of MyoD and desmin [47] but fails to complete terminal differentiation [48, 49]. These data indicate that oncogenesis of FN-RMS may result from not only cell growth, but also differentiation failure caused by the dysregulated function of HMGA2.

Along these lines, we examined whether netropsin might have an antitumor effect in FN-RMS. Indeed, netropsin inhibited the proliferation of FN-RMS in a dose-dependent manner (Fig. 6). Netropsin is a minor groove-binding protein targeting AT-rich DNA. Such a minor groove of AT-rich DNA sequences is also a binding site for HMGA family proteins. Therefore, netropsin competes with the HMGA family proteins HMGA1 and HMGA2 for DNA binding and interferes with their function [14, 15]. Lau et al. [50] showed that netropsin inhibited HMGA1-expressing medulloblastoma cell growth in vitro and in vivo, with a reduction in HMGA1-targeted RNA promoter activity and expression. The IC_50_ value of netropsin in that study was in the micromolar order, as in our findings. These results make this minor grove inhibitor a promising antitumor agent for HMGA2-expressing FN-RMS. The limitation of this finding is that it is not clear whether the antitumor effect of netropsin on FN-RMS cells is due to specific inhibition of HMGA2. Investigation of HMGA2–target gene expression could prove its specificity. However, the HMGA2 target gene contributing to the proliferation of FN-RMS cells is still unknown.

## Conclusions

In conclusion, this study has yielded two important observations. First, high HMGA2 expression leads to FN-RMS oncogenesis and represents a potentially attractive therapeutic target in FN-RMS. Second, netropsin, a small-molecule minor groove-binding protein, is a promising agent for the treatment of FN-RMS patients.

## Data Availability

The datasets used and analyzed during the current study are available from the corresponding author on reasonable request.

## Abbreviations

RMS: Rhabdomyosarcoma
ERMS: Embryonal rhabdomyosarcoma
ARMS: Alveolar rhabdomyosarcoma
COG: Children’s Oncology Group
FN-RMS: Fusion gene-negative rhabdomyosarcoma
FP-RMS: Fusion gene-positive rhabdomyosarcoma
HMGA: High mobility group A
RT-PCR: Reverse transcription-polymerase chain reaction
GAPDH: Glyceraldehyde-3-phosphate dehydrogenase
SDS-PAGE: Sodium dodecyl sulfate–polyacrylamide gel electrophoresis
PBS: Phosphate-buffered saline
PBST: Phosphate-buffered saline with Tween 20
PI: Propidium iodide
MHC: Myosin heavy chain
MSCV: Murine stem cell virus
